# Metacognitive insight into cognitive performance in Huntington’s disease gene carriers

**DOI:** 10.1136/bmjno-2022-000268

**Published:** 2022-04-05

**Authors:** Samuel RC Hewitt, Alice J White, Sarah L Mason, Roger A Barker

**Affiliations:** 1 John Van Geest Centre for Brain Repair, Department of Clinical Neuroscience, University of Cambridge, Cambridge, Cambridgeshire, UK; 2 Max Planck UCL Centre for Computational Psychiatry and Ageing Research, University College London, London, UK; 3 Wellcome Centre for Human Neuroimaging, University College London, London, UK

**Keywords:** HUNTINGTON'S, COGNITION, COMPUTATIONAL PSYCHIATRY

## Abstract

**Objectives:**

Insight is an important predictor of quality of life in Huntington’s disease and other neurodegenerative conditions. However, estimating insight with traditional methods such as questionnaires is challenging and subjected to limitations. This cross-sectional study experimentally quantified metacognitive insight into cognitive performance in Huntington’s disease gene carriers.

**Methods:**

We dissociated perceptual decision-making performance and metacognitive insight into performance in healthy controls (n=29), premanifest (n=19) and early-manifest (n=10) Huntington’s disease gene carriers. Insight was operationalised as the degree to which a participant’s confidence in their performance was informative of their actual performance (metacognitive efficiency) and estimated using a computational model (HMeta-d’).

**Results:**

We found that premanifest and early-manifest Huntington’s disease gene carriers were impaired in making perceptual decisions compared with controls. Gene carriers required more evidence in favour of the correct choice to achieve similar performance and perceptual impairments were increased in those with manifest disease. Surprisingly, despite marked perceptual impairments, Huntington’s disease gene carriers retained metacognitive insight into their perceptual performance. This was the case after controlling for confounding variables and regardless of disease stage.

**Conclusion:**

We report for the first time a dissociation between impaired cognition and intact metacognition (trial-by-trial insight) in the early stages of a neurodegenerative disease. This unexpected finding contrasts with the prevailing assumption that cognitive deficits are associated with impaired insight. Future studies should investigate how intact metacognitive insight could be used by some early Huntington’s disease gene carriers to positively impact their quality of life.

**Key messages**

**What is already known on this topic**

- Huntington’s disease (HD) gene carriers can underestimate their impairments when asked to explicitly reflect on them. However, self-reports about cognition and daily life are confounded by many factors.

**What this study adds**

- This study shows that insight into cognitive performance remains intact in high functioning, premanifest and early-manifest HD gene carriers, even when the cognitive performance itself is impaired.

**How this study might affect research, practice or policy**

- Insight into impaired cognition is a putative mechanism for increased anxiety and depression commonly reported by HD gene carriers, which should be further investigated. Psychological interventions which use metacognition may help some early HD gene carriers exploit this intact skill to benefit their quality of life.

## Introduction

Huntington’s disease (HD) is a neurodegenerative disorder caused by a CAG expansion in exon 1 of the Huntingtin gene.^1^ HD gene carriers are currently diagnosed with manifest disease when abnormal movements emerge, but true disease onset begins years earlier.^2^ The cognitive features of HD develop in the premanifest stage and include impaired executive cognition (planning, reasoning, working memory and attention^3^), psychomotor processing speed, visuospatial functions and emotion recognition.^4^ Patients tend to perceive their abilities differently from their carers, typically underestimating their impairments when asked to explicitly reflect on them.^5^ We refer to this as global insight, and it is thought that HD patients become increasingly impaired as disease burden increases.

However, studies of global metacognitive insight such as those which rely on self-report are subjected to several confounding influences, which limit their interpretability. This is because global insight is a complex concept, which is influenced by many individual differences. For instance, systematic response biases (eg, optimism), personality dimensions or temporary psychological states (eg, trait-anxiety or stress) and other critical cognitive functions (eg, episodic memory) can all affect the way that patients report on themselves. Here, we specify metacognitive insight as the accuracy of reflection on performance in a cognitive task (ie, insight into task performance on a trial-by-trial basis). This has been referred to as local metacognition and is distinct from global insight. Global insight is hierarchically more abstract, spans longer timescales and captures how we feel about performance broadly, for example, across an entire task, a cognitive domain or in daily life.^6^

Local metacognitive insight has been associated with neural substrates, which are also affected early on in HD. For example, in healthy controls, metacognition has been associated with increased anterior and medial prefrontal cortex activity^7^ and altered hippocampal myelination.^8^ Premanifest HD gene carriers exhibit grey matter loss in the prefrontal cortex^9^ and hippocampal dysfunction is reported with late premanifest and manifest HD.^10^ However, metacognitive insight, as defined here, has not been explicitly tested in HD.

To measure metacognitive insight, we asked participants to report their confidence in their decision-making performance after each trial of a taxing visual perception task. Objective decision-making performance was controlled across participants by adjusting the difficulty of the task based on their response accuracy. We used an established computational model to estimate metacognitive insight into that performance from participant’s confidence ratings.^11^ This allowed us to dissociate cognitive (perceptual decision-making) performance from metacognitive insight into performance across premanifest and early-manifest HD and age-matched and sex-matched healthy controls. We hypothesised that HD gene carriers would show impairments in perceptual performance. We further hypothesised that this would be compounded by a reduction in metacognitive insight into performance. We predicted that both these impairments would be significantly greater in those with early-manifest disease.

## Methods

### Participants

Sixty-three participants completed this study: 14 patients with early-manifest HD, 20 premanifest gene carriers and 29 healthy controls between September 2019 and November 2020. All HD gene carriers were genetically confirmed (CAG ≥36). Patients were defined as having early-manifest disease when they had a Unified Huntington’s Disease Rating Scale (UHDRS) total motor score >5.^12 13^ The groups were matched for age and sex. Inclusion criteria were Mini-Mental State Examination (MMSE) Score >26 (normal range) and UHDRS initiation and saccade velocity total scores less than or equal to 1 (indicating minimal impairment in one domain only; maximum score is 16). Therefore, all included participants with gene-positive HD had no global cognitive or saccadic impairments as detected during examination by an experienced Consultant Neurologist (RAB). Exclusion criteria were any significant comorbid psychiatric or neurological diagnoses. Participants with HD were recruited from the HD Clinic at the University of Cambridge and Cambridge Universities Hospitals NHS (National Health Service) Foundation Trust. Controls were recruited from the local community. Clinical data were collected and managed using REDCap (Research Electronic Data Capture) electronic data capture tools hosted at the University of Cambridge.^14^ Anonymised data used in this study are available online.^15^This study is reported in accordance with the STROBE reporting checklist.^16^

### Stimuli and procedure

**Figure 1:**
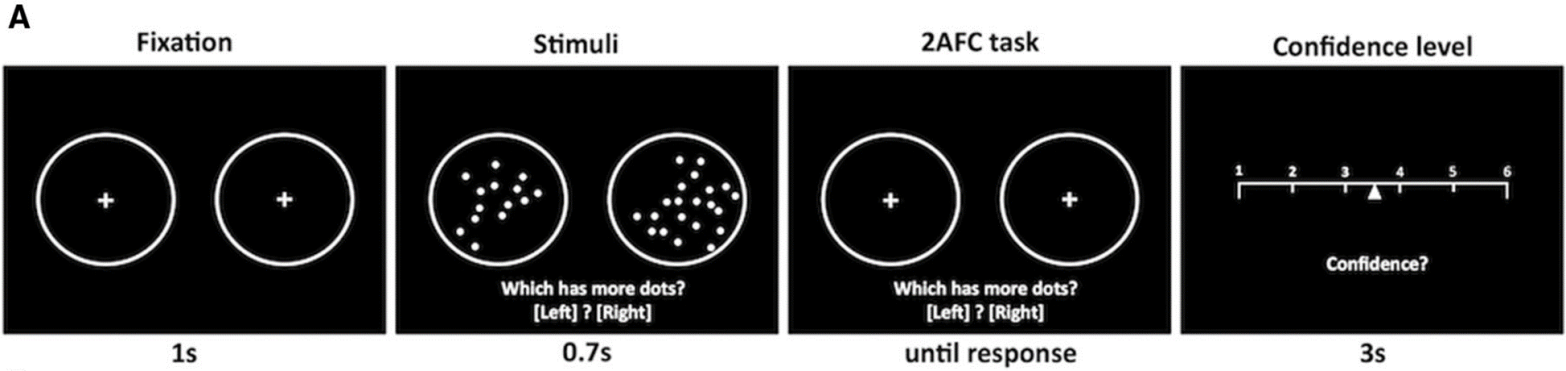
Meta-dots task. Participants make an alternative-forced choice judgement (2-AFC) about which of the two stimuli (circles) contain more dots. This is immediately followed by a confidence rating on each trial.

**Figure 2:**
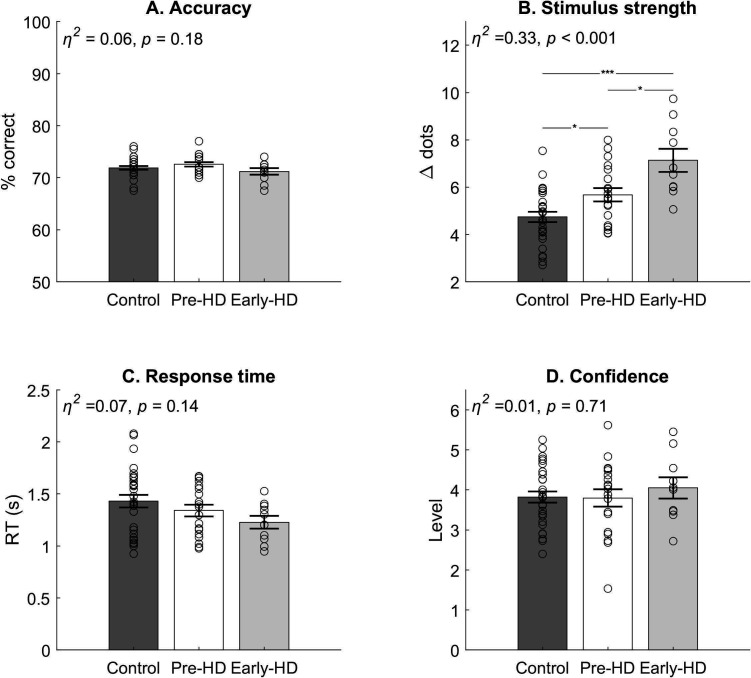
Behavioural data. (A) Accuracy was controlled across the groups at approximately 71%. (B) Stimulus strength (Δ dots) was significantly increased in the early-manifest group, compared with both groups, and also in the premanifest group compared with the control group. (C) No significant difference in mean response time. (D) No significant difference in mean confidence. Bars=mean ± standard error of the mean. Circles=individual mean values. *Bonferroni corrected p<0.05. ***Bonferroni corrected p<0.001. *η^2^*=ETA squared effect size. Early-HD, early-manifest Huntington’s disease; Pre-HD, premanifest Huntington’s disease.

We employed a task previously used to separately assess perceptual decision-making and metacognition,^17^ implemented in MATLAB using Psychtoolbox.^18^ The code used to run the task is available online.^19^ Participants were required to make an alternative forced-choice judgement about which of two briefly presented (0.7 s) circles contained more dots, for which there was no response time limit. One of the two circles contained 50 dots while the other circle contained a number bounded between 1 and 100. On each trial, this was followed by a confidence rating which had to be made within 4 s of the confidence scale being shown. All stimuli were high contrast (white on black; figure 1). A one-up two-down staircase procedure equated performance across participants based on response accuracy by manipulating the stimulus strength (Δ dots) such that performance was constant (~71%, figure 2A). The staircase procedure was initiated during a practice phase which provided feedback on decision accuracy. Feedback was not given after the practice. The experiment was divided into 8 blocks of 25 trials, separated by a break of length determined by the participant.

Participants also completed the Hospital Anxiety and Depression Scale (HADS), MMSE and the National Adult Reading Test, which was used to calculate predicted verbal intelligence quotient (IQ).

### Metacognitive insight

We used metacognitive efficiency (M-ratio) as an index for metacognitive insight across premanifest HD, early-manifest HD and healthy controls. M-ratio is an established marker of metacognition based on signal detection theory.^20^ M-ratio describes how much of the available signal (ie, a participant’s perceptual sensitivity, *d’*) is captured by their confidence about their performance on each trial. Specifically, M-ratio is the ratio between metacognitive sensitivity (*meta-d’*) and perceptual sensitivity (*d’*). As such, this method controls for differences in perceptual ability as well as response biases (eg, repeatedly high confidence) and is well suited to compare metacognitive insight in clinical groups. An M-ratio of 1 would represent optimal sensitivity to perceptual performance. If M-ratio <1, there is some noise in the confidence ratings, such that the individual does not exploit all the available perceptual signal for their metacognitive judgement. If M-ratio >1, this implies that the individual can draw on additional information about themselves or the task (beyond the available perceptual signal) when reporting on their performance.^21^ We estimated M-ratio using a hierarchical modelling approach implemented in an openly available MATLAB toolbox (HMeta-d’^11^). This toolbox is a Bayesian extension of the original metacognitive efficiency model^22^ and provides robust parameter estimates in the face of uncertainty inherent in clinical groups of small sample size and relative heterogeneity.^11^

### Perceptual decision-making

We also complimented the analysis of perceptual (first-order cognitive) decisions by estimating latent components of the decision-making process using the hierarchical drift diffusion model (HDDM).^23^ Like HMeta-d’, HDDM is particularly well suited to clinical research studies because it captures sources of uncertainty in the data (eg, small group size and heterogeneous group features) in the form of posterior probability distributions of the parameter estimates. HDDM uses the choice and reaction time data to calculate latent parameters, which estimate how individuals made perceptual decisions during the task (online supplemental figure 1). This was implemented in the openly available HDDM python toolbox (V.0.8.0). Details of the implementation process, model comparison and validation are available in online supplemental information.

10.1136/bmjno-2022-000268.supp1 Supplementary data

### Statistical power

We powered this study *a priori* to detect a difference in metacognitive insight based on the effect size obtained by Fleming *et al*^17^ as there are no published findings in HD. Their study detected differences across two clinical groups and controls using the same task and analysis method. We estimated the effect size (Cohen’s *f=*0.53, α=0.05, two-tailed) based on reported means. This revealed that a total sample size of 39 was required to achieve power of 0.8.

## Results

### Participant demographics

Five participants were excluded prior to the analysis; four early-manifest HD patients were excluded due to saccadic impairment and one individual with premanifest HD was excluded due to a technical error while they completed the task. Included participants (N=58) were well-matched for age and sex across the groups (table 1). All participants had MMSE Scores in the normal range, but the early-manifest HD group had lower scores (H(2)=10.5, p=0.005). Premorbid verbal IQ was significantly lower in the premanifest and early-manifest groups (F(2, 54)=5.2, p=0.009). Linear regression models were later used to understand if these differences were related to metacognitive efficiency. The early-manifest group had lower total functional capacity scores than premanifest HD patients, as expected (W=164, p<0.01). Three of the early-manifest patients and one premanifest gene carrier were taking low-dose Olanzapine (2.5–5 mg/day) for clinical reasons relating to their condition.

**Table 1:** Participant demographics

	Premanifest HD (N=19)	Early-manifest HD (N=10)	Control (N=29)	Test statistic	P value
Age				1.5	0.226 (1)
- Mean	47.8	55.9	51.6		
- Range	28.7–75.4	37.2–67.0	29.3–73.4		
Sex, female	11 (57.9%)	7 (70.0%)	12 (41.4%)	2.9	0.238 (2)
MMSE				10.5	**0.005 (3**)
- Mean	29.7	28.6	29.7		
- Range	28.0–30.0	26.0–30.0	28.0–30.0		
Premorbid verbal IQ				5.2	**0.009 (1**)
- Mean	113.5*	111.6	118.1		
- Range	100.0–127.0	104.0–124.0	107.0–127.0		
UHDRS total motor				4	**<0.001(4**)
- Mean	2.3	14.7	–		
- Range	0.0–12.0†	6.0–26.0	–		
TFC				164	**<0.001(4**)
- Mean	12.8	11.4	–		
- Range	11.0–13.0	10.0–13.0			

Groups were matched for age and sex. Groups had clinically normal, yet statistically different general cognitive and verbal IQ scores. The premanifest and early-manifest patients were different in their total UHDRS motor scores and functional capacity, as expected. Bolded p values indicate significance at p<0.05. (1) Linear model ANOVA, (2) Pearson’s χ^2^ test, (3) Kruskal-Wallis one way ANOVA, (4) Wilcoxon Mann-Whitney Rank Sum test.

*One premanifest individual did not complete the National Adult Reading Test for verbal IQ.

†One premanifest individual had an unusually high motor score due to an unrelated hand injury.

ANOVA, Analysis of variance; MMSE, Mini-Mental State Examination; TFC, Total Functional Capacity; UHDRS, Unified Huntington’s Disease Rating Scale.

### Behavioural analysis

To assess behavioural performance, we compared mean accuracy (% correct), stimulus strength (Δ dots), response time and confidence ratings using one-way analysis of variance (ANOVA) or Kruskal-Wallis tests as non-parametric equivalent (see online supplemental information for methods of statistical test selection). The staircase procedure successfully matched accuracy (% correct; figure 2A) across the groups (H(2, 55)=1.91, p=0.38, η^2^=0.06). However, the mean stimulus strength to achieve that performance differed significantly between the groups (F(2, 55)=13.85, p<0.001, η^2^=0.33; figure 2B). Pairwise comparison with Bonferroni correction method showed that patients with early-manifest HD (mean=7.13 ± SEM=0.4) completed the task with significantly greater stimulus strength (ie, reduced difficulty level) compared with the premanifest group (mean=5.68 ± SEM=0.29; 95% CIs of mean difference=1.25 to 3.53, adjusted p<0.001) and also compared with healthy controls (mean=4.74 ± SEM=0.23; 95% CIs of mean difference 0.24 to 2.67, adjusted p=0.014). Furthermore, the premanifest group performed with a significantly greater stimulus strength than the control group (95% CIs of mean difference 0.02 to 1.86, adjusted p*=*0.043). This shows that individuals with premanifest and early-manifest HD were impaired in making perceptual decisions compared with healthy controls. There were no significant differences in mean response time (F(2, 55)=2.03, p=0.14, η^2^=0.07; figure 2C). However, the trend towards reduced response time with manifest HD was further explored using the HDDM. There were also no differences in confidence level across the groups (F(2, 55)=0.34, p=0.71, η^2^=0.01; figure 2D). This confirms that all participants were able to execute the perceptual decision and use the confidence scale as instructed. In addition, task accuracy was also matched across the groups throughout the entire experiment. There were no differences in accuracy across the eight task blocks (F(7, 440)=0.59, p=0.77, η^2^_p_ = 0.01), and no interaction effect of group by block (F(14, 440)=1.02, p=0.43, η^2^_p_ = 0.03).

### Perceptual decision-making model

We compared a limited number of regression models in order to determine the best-fitting HDDM to perceptual reaction time data. The best-fitting model (lowest Bayesian Predictive Information Criterion (BPIC) and Deviance Information Criterion (DIC); online supplemental table 1) was characterised by a regression in which drift rate was modulated by group and stimulus strength, their interaction, and decision threshold was modulated by group. Model parameters were reproducible (online supplemental table 2) and simulated reaction time data based on these accurately reproduced response times observed in our participants, including the trend towards faster response times with manifest HD (online supplemental figure 2). Analysis of the posterior distributions of model parameters showed that healthy controls responded to stronger evidence (Δ dots, z-scored within subjects) by significantly increasing their rate of evidence accumulation (drift rate), compared with both premanifest (*P*<0.001) and early-manifest gene carriers (*P*<0.001) who did not differ from eachother (*P*=0.34). Furthermore, premanifest gene carriers set significantly lower decision thresholds for evidence accumulation than controls (*P*<0.001), an impairment which was significantly greater in those with early-manifest disease (*P*<0.001, online supplemental figure 3).

### Metacognitive insight

M-ratio for each group was estimated separately and a higher value indicated better metacognitive insight. To assess if meaningful differences existed between the groups, we calculated 95% high-density intervals (HDI) of differences between two distributions in pair-wise comparisons and compared the resulting difference distribution with 0. If the 95% HDI excluded 0, we considered this to be a meaningful (significant) difference.

**Figure 3:**
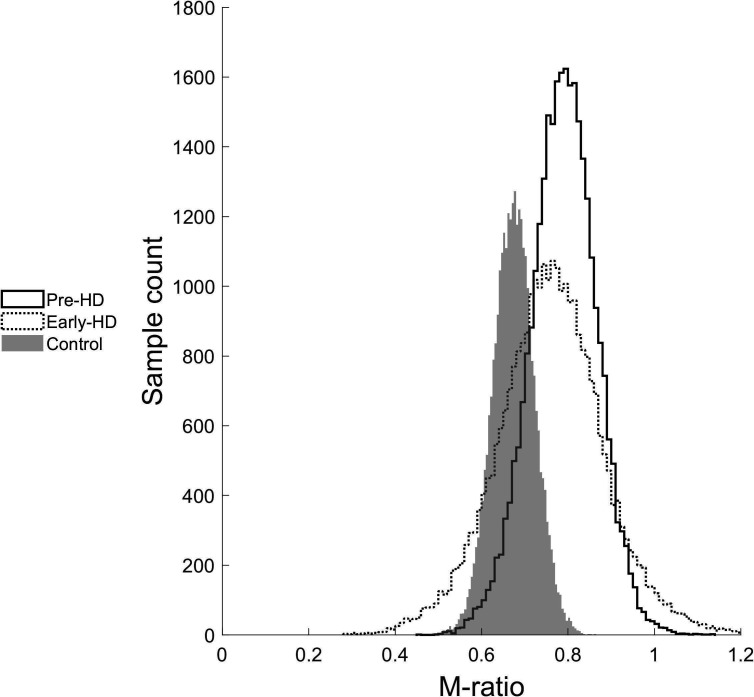
M-ratio sample estimates across the groups. There is significant overlap in the distributions indicating that gene carriers showed similar metacognitive insight to controls. Early-HD, early-manifest Huntington’s disease; Pre-HD, premanifest Huntington’s disease.

**Figure 4:**
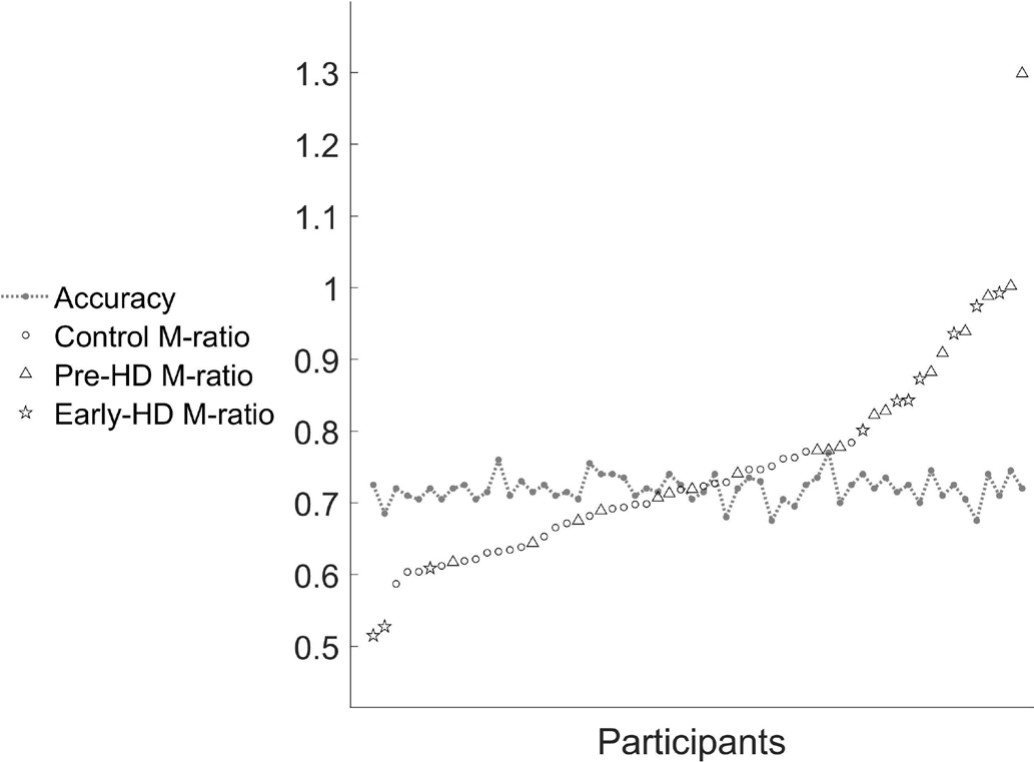
Individual mean accuracy (proportion correct) controlled at approximately 0.71 and mean M-ratio estimates. Each participant is a point on the X-axis. Early-HD, early-manifest Huntington’s disease; Pre-HD, premanifest Huntington’s disease.

There was no difference in metacognitive efficiency (M-ratio) between healthy controls (M: 0.68) and premanifest HD gene carriers (M: 0.82; p=0.1, 95% HDIs: −0.095 to +0.388). There was also no difference between the early-manifest HD gene carriers (M: 0.79) and the control group (*P*=0.25, 95% HDIs: −0.282 to +0.475). M-ratio was not reduced with greater disease burden, since early-manifest HD gene carriers did not significantly differ from the premanifest group (*P=*0.59, 95% HDIs: −0.458 to +0.34; figure 3). Mean M-ratio and response accuracy for individual participants are plotted in figure 4.

**Figure 5:**
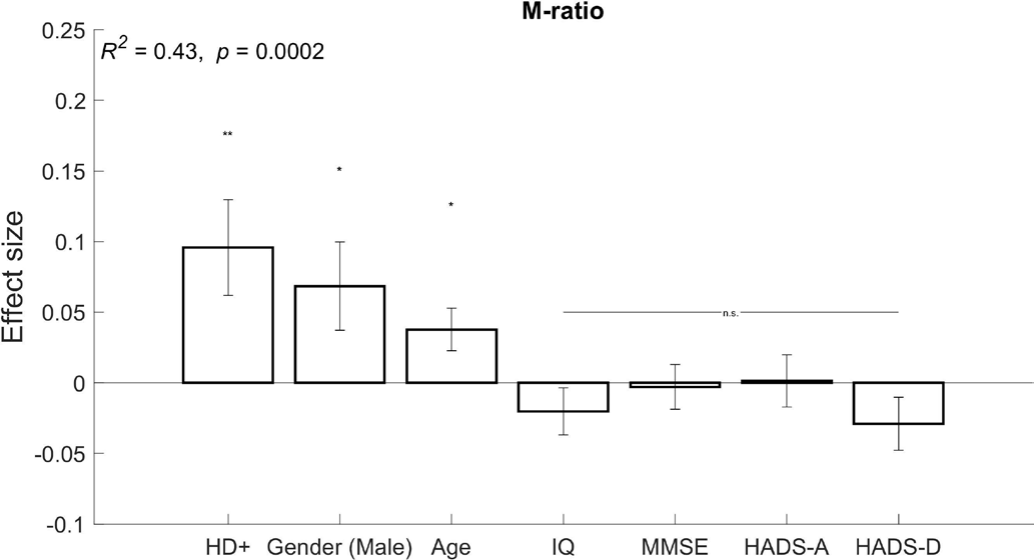
Linear regression coefficients for M-ratio (metacognitive efficiency) with independent predictors: Huntington’s disease (HD) gene status, age, gender, IQ, Mini-Mental State Examination (MMSE) score, Hospital Anxiety and Depression Score (HADS)-Anxiety and HADS-Depression. n.s, not significant, *p<0.05 and **p<0.01. Error bars indicate standard error of the mean.

To understand the contribution of individual differences, we conducted a post hoc regression analysis in which metacognitive parameters (‘M-ratio’, ‘metacognitive sensitivity’, ‘perceptual sensitivity’, ‘confidence’) were dependent variables. Predictors were HD gene status and several clinical covariates (age, gender, IQ, MMSE score, HADS-Anxiety score, HADS-Depression score). Continuous predictor variables were z-scored prior to the regression. Significance level for each regression model was adjusted using Bonferroni correction for the number of dependent variables (0.05/4=0.0125). This confirmed the previous finding that HD gene carriers had intact metacognitive insight. A genetic diagnosis of HD was a significant predictor of improved metacognitive efficiency (*β* =+0.096, p=0.007) after controlling for confounding individual differences (*R^2^*=0.43, p<0.001; figure 5). We found that HD gene status (*β* =+0.114, p=0.003) was also a significant positive predictor of metacognitive sensitivity (*R^2^*=0.4, p<0.001) but did not predict perceptual sensitivity (*R^2^*=0.06, p=0.83). Since metacognitive efficiency is simply the ratio between metacognitive and perceptual sensitivity (*meta-d*’/*d*’), this confirms that intact metacognitive efficiency in HD gene carriers was driven by *increased metacognitive* sensitivity (*meta-d*’) and not *reduced perceptual* sensitivity (*d’*). Confidence itself was not directly associated with HD gene status, age, gender, IQ, cognition, anxiety or depression (R^2^*=*0.22, p*=*0.09).

## Discussion

We report two novel findings about HD. First, there is a deficit in perceptual decision-making that can be seen in the premanifest stage of the condition and gets worse in manifest disease, indicating that it is a product of the disease process rather than a genotype effect that is stable between disease stages. Second, despite impaired perceptual decision-making performance, both premanifest and manifest HD gene carriers demonstrated similar metacognitive insight into their performance as the control group. In summary, we report a dissociation between impaired first-order cognition and intact, second-order, metacognition (trial-by-trial insight) in premanifest and early-HD gene carriers.

HD gene carriers required the perceptual decisions to be made objectively easier in order to perform as well as controls. Furthermore, a computational model revealed that this was underlined by impairments in evidence accumulation and reduced decision thresholds. This was expected, as early-manifest HD patients are impaired in the identification of ambiguous shapes and objects^24^ and both premanifest and manifest gene carriers show impairments in the recognition of faces and emotions.^25–27^

In contrast, we predicted that metacognitive insight would be impaired in HD gene carriers but found evidence to reject this hypothesis. Posterior distributions of metacognitive efficiency across all three groups did not differ. In a post hoc analysis, having the HD gene was a significant predictor of improved metacognitive efficiency after controlling for the influence of age, gender, IQ, cognition, anxiety and depression. This was due to increased metacognitive sensitivity in HD gene carriers and not reduced perceptual sensitivity. Age and gender were also significant predictors of metacognitive efficiency but IQ, cognition, anxiety and depression were not (figure 5).

A possible explanation for intact metacognitive performance (despite impaired perceptual decision-making) is that a genetic diagnosis of HD induces a prior belief of current or future impairment and this leads to increased vigilance to performance—either consciously or subconsciously. In line with this, gene carriers and their families often report ‘symptom hunting’ and it is possible that trial-by-trial metacognitive insight is attuned by this over time. However, we found no evidence of a negative confidence bias in gene carriers (ie, generally lower confidence; figure 2D). Although intact metacognitive insight into HD gene carriers was contrary to our hypothesis, other recent studies have identified performance improvements associated with HD gene expansion. For example, Huntingtin gene expansion in low pathological ranges is associated with improved cognitive test scores and superior IQ performance in far-from-onset gene carriers.^28 29^

Intact metacognitive insight despite (impaired) cognitive performance in premanifest and early-HD is of clinical interest because it may be relevant to subjective well-being and mental health.^6^ HD causes a wide range of psychological difficulties, but the literature on psychological interventions for people affected by HD is extremely limited.^30^ A recent feasibility study has shown that mindfulness-based cognitive therapy (which exploits metacognition) can be beneficial to individuals with premanifest HD.^31^ Our finding that HD gene carriers retain good metacognitive insight (despite deficits in cognitive performance), further indicates that psychological therapies designed to apply this positively, may help maintain psychological well-being following a genetic diagnosis of HD.

### Limitations

The aim of this study was to assess whether local (trial-by-trial) metacognitive insight into cognitive performance is affected in the early stages of the HD disease process. We have shown that in relatively high functioning HD gene carriers, metacognitive insight into cognitive performance is intact even though the performance itself is impaired. However, these findings relate only to HD gene carriers who have *not* developed marked functional and cognitive impairments. Metacognitive insight may well decline as HD progresses. Consistent with this, there was increased uncertainty in the M-ratio for the early-manifest HD group; the posterior distribution is wider, with longer tails (figure 3). This is likely due to the smaller sample size and greater heterogeneity of this group.

Second, changes in metacognitive performance may still occur early in HD in other cognitive domains or over different timescales (eg, global insight). Research into metamemory in Alzheimer’s dementia has shown that local (ie, trial-by-trial) metacognitive estimates are intact but global self-estimates are altered.^32^ Future studies should consider the progression between (early stage, intact) local and (later stage, possibly impaired) global metacognitive insight in HD gene carriers.

We did not include medication effects in our analyses. Dopamine is well known to affect cognition^33^ and manifest HD patients are often prescribed dopamine antagonists to help with the disease features, but these can increase the rate of cognitive decline.^34^ However, only 4 of 29 (13.8%) gene carriers in this study were taking antidopaminergic medication, and all at low dose, so the pattern of findings cannot be explained by this.

## Conclusion

By dissociating perception and metacognition in HD, we show that perceptual decision-making impairments exist in HD gene carriers without any other obvious symptoms or signs. However, metacognitive insight into cognitive performance remains intact, even in those who have progressed to manifest disease. Low-level perceptual issues, which appear early in the disease, may drive higher order cognitive deficits that are often seen in the HD clinic. However, since metacognition is closely related to well-being and quality of life, clinicians and researchers should investigate how to exploit the metacognitive insight that some HD gene carriers can demonstrate.

## Data Availability

Data are available in a public, open access repository. Anonymised clinical and demographic information, task data, model outputs and scripts used for analysis are available on Github (https://github.com/samrchewitt/HD_perception_metacognition). These are shared under Creative Commons Zero v1.0 Universal license.
